# Dataset of tear film cytokine levels in dry eye disease (DED) patients with and without HIV infection

**DOI:** 10.1016/j.dib.2016.11.027

**Published:** 2016-11-26

**Authors:** Praveen Kumar Balne, Rupesh Agrawal, Veonice Bijin Au, Bernett Lee, Eileen Loo, Louis Tong, Arkasubhra Ghosh, Stephen C. Teoh, John Connolly, Petrina Tan

**Affiliations:** a National Healthcare Group Eye Institute, Tan Tock Seng Hospital, Singapore; b Singapore Eye Research Institute, Singapore; c Institute of Cell and Molecular Biology, Singapore; d Duke-NUS Medical School, Singapore; e National University of Singapore, Singapore; f GROW Research Laboratory, Narayana Nethralaya Foundation, India; g Eagle Eye Centre, Singapore; h Jurong General Hospital, Singapore

**Keywords:** Cytokines, Ocular surface, Dry eye disease, HIV, GRO

## Abstract

The tear film cytokine profiling data in this article was obtained from a prospective case-control study with a sample size of 34 dry eye disease (DED) patients with HIV infection and 32 DED patients without HIV infection, see “A distinct cytokines profile in tear film of dry eye disease (DED) patients with HIV infection” (R. Agrawal, P.K. Balne, A. Veerappan, V.B. Au, B. Lee, E. Loo, A. Ghosh, L. Tong, S.C. Teoh, J. Connolly, P. Tan, 2016) [1]. Tear samples were collected from all the subjects using Schirmer׳s strips and cytokine profiling was done using the Luminex bead based multiplex assay with a panel of 41 analytes. The cytokine level differences in each group of subjects were analyzed using logistic regression models.

**Specifications Table**

**Table:** 

Subject area	*Biology*
More specific subject area	*Tear film cytokines in Dry Eye Disease patients*
Type of data	*Table*
How data was acquired	*FlexMAP 3D (Luminex*®*) platform using the Milliplex*® *MAP human cytokine/chemokine magnetic bead panel -1 kit*
Data format	*Raw*
Experimental factors	*Tear samples collected from DED patients with and without HIV infection and the concentrations of 41 cytokines in each sample were measured using Luminex bead based multiplex assay*
Experimental features	*FlexMAP 3D (Luminex*®*) platform with Milliplex*® *MAP human cytokine/chemokine magnetic bead panel -1 kit (Millipore, USA) was used for Tear film cytokine profiling*
Data source location	*Singapore*
Data accessibility	*Data is with this article*

**Value of the data**

• This is the first dataset of tear film cytokines in HIV patients with dry eye disease and its comparison with dry eye disease patients without HIV infection [1].
• The data show the significantly increased levels of cytokines epidermal growth factor (EGF) and IFN-gamma-inducible protein 10 (IP-10, CXCL10) and decreased levels of growth-regulated oncogene (GRO) in HIV patients with DED and explains the possible role of these 3 cytokines in HIV associated ocular inflammation in DED pathogenesis [1].
• None of the cytokine concentrations in HIV patients with DED correlated with the clinical parameters indicate that the levels of these 3 cytokines in these patients are independent of clinical severity of the disease and mainly mediated by HIV [1].
• The data presented in this report will help researches to study the disease pathogenesis and mechanisms of DED associated with HIV infection.

## 1 Data

The tear film cytokine profiles data obtained from DED patients with and without HIV infection (Table 1) using Luminex bead based multiplex assay with a panel of 41 analytes reveled different cytokine signatures in each group [1]. The clinical data (Table 2) represents the severity of the DED was evaluated using different clinical tests following the guidelines of Dry Eye Workshop 2007.

## 2 Experimental design, materials and methods

Tear samples were collected from 34 DED patients with HIV infection and 32 DED patients without HIV infection with prior informed consent [1]. All the subjects presented to the clinic with the dry eye related symptoms were evaluated by clinical presentation according to the International Dry Eye Workshop, 2007 [2]. Dry eye disease related tests (Table 2) including tear film break up time (TBUT), Schirmer׳s test, corneal staining, conjunctival staining, conjunctival injection measurements and examination of lid margin changes and meibomian gland were done for all the subjects for the final diagnosis of the DED. Western blot assay (Diagnostic Biotechnology HIV Blot 2.2) was done for the confirmation of HIV diagnosis and the tear samples were collected from all the subjects using Schirmer׳s strips. Tear fluid was eluded from Schirmer׳s strips by incubating with 30 µl of assay buffer at room temperature for 5 min followed by 1 min centrifugation at 20,000 rpm and the eluded tear samples were stored at −80 °C freezer until cytokine analysis [3]. The frozen tear samples were thawed on ice and centrifuged for 5 min at 3000 rpm and cytokine profiling was done for 41 analytes (Table 1) in each sample by Luminex bead based multiplex assay using Milliplex^®^ MAP human cytokine/chemokine magnetic bead panel −1 kit (Millipore, USA) following the manufacturer׳s instructions. In each group the cytokine differences were analyzed using logistic regression models and the cytokine levels were correlated with clinical parameters.

## Appendix A Supplementary material

Table 1: Concentrations of 41 cytokines in the tear film of DED patients with and without HIV infection.

.

Table 2: Clinical parameters of DED patients with and without HIV infection.

## Footnotes

Appendix A Supplementary data associated with this article can be found in the online version at doi:10.1016/j.dib.2016.11.027.
